# Insight into telomere regulation: road to discovery and intervention in plasma drug-protein targets

**DOI:** 10.1186/s12864-024-10116-5

**Published:** 2024-03-02

**Authors:** Kaixi Ding, Juejue Zhangwang, Ming Lei, Chunping Xiong

**Affiliations:** 1 School of Clinical Medicine, Chengdu University of Traditional Chinese Medicine, Chengdu, 610075 China; 2 Hospital of Chengdu University of Traditional Chinese Medicine, Chengdu, 610075 China

**Keywords:** Telomere length, Protein expression regulatory loci, Mendelian randomization, Target proteins, Causal associations

## Abstract

**Background:**

Telomere length is a critical metric linked to aging, health, and disease. Currently, the exploration of target proteins related to telomere length is usually limited to the context of aging and specific diseases, which limits the discovery of more relevant drug targets. This study integrated large-scale plasma cis-pQTLs data and telomere length GWAS datasets. We used Mendelian randomization(MR) to identify drug target proteins for telomere length, providing essential clues for future precision therapy and targeted drug development.

**Methods:**

Using plasma cis-pQTLs data from a previous GWAS study (3,606 Pqtls associated with 2,656 proteins) and a GWAS dataset of telomere length (sample size: 472,174; GWAS ID: ieu-b-4879) from UK Biobank, using MR, external validation, and reverse causality testing, we identified essential drug target proteins for telomere length. We also performed co-localization, Phenome-wide association studies and enrichment analysis, protein-protein interaction network construction, search for existing intervening drugs, and potential drug/compound prediction for these critical targets to strengthen and expand our findings.

**Results:**

After Bonferron correction (*p* < 0.05/734), RPN1 (OR: 0.96; 95%CI: (0.95, 0.97)), GDI2 (OR: 0.94; 95%CI: (0.92, 0.96)), NT5C (OR: 0.97; 95%CI: (0.95, 0.98)) had a significant negative causal association with telomere length; TYRO3 (OR: 1.11; 95%CI: (1.09, 1.15)) had a significant positive causal association with telomere length. GDI2 shared the same genetic variants with telomere length (coloc.abf-PPH 4 > 0.8).

**Conclusion:**

Genetically determined plasma RPN1, GDI2, NT5C, and TYRO3 have significant causal effects on telomere length and can potentially be drug targets. Further exploration of the role and mechanism of these proteins/genes in regulating telomere length is needed.

**Supplementary Information:**

The online version contains supplementary material available at 10.1186/s12864-024-10116-5.

## Introduction

Telomere length is an essential indicator of individual cellular health, and its close association with health outcomes and aging has received much attention. As DNA sequences at the ends of chromosomes, telomeres protect chromosomes from loss [1]. Telomere structure and function are critical for maintaining a normal life cycle [1]. Various factors, such as genetic factors, environmental exposures, disease states, and biological age, can influence the maintenance and changes in telomere length in individuals [2]. Longer telomere length has been associated with reduced mortality due to all-cause mortality and cardiovascular disease-specific mortality, reduced risk of coronary heart disease, and increased healthy lifespan and better health in centenarians [3–6]. Conversely, shorter telomere length increases the risk of all-cause mortality and several diseases, including aplastic anemia, pulmonary fibrosis, gastric cancer, diabetes, and age-related diseases [7–10]. Evidently, telomere length is vital in marking aging and predicting health and disease.

Researchers have recently uncovered the link between specific gene- or protein-related telomere length regulatory effects and certain diseases. A Mendelian randomization(MR) study found that a putative target of simulated metformin, GPD1-induced reduction in HbA1c, was positively associated with longer leukocyte telomere lengths [11]. EGFR enhances telomerase activity (affecting telomere lengths) by potentiating the transcription of TERT, which correlates with the differentiation grade and prognosis of non-small-cell lung cancer [12]. The Shelterin Complex consists of six proteins: TRF1, TRF2, POT1, RAP1, TIN2 and TPP1. Its dysfunction or defects may shorten telomeres and promote aging [13]. Upregulation of ETS transcription factors involved in the reactivation of telomerase can diminish the efficacy of BRAF inhibitors in patients with BRAF-mutant pediatric gliomas [14]. In the future, an important research direction is to develop specific drug targets to intervene in telomere length, leading to the treatment of diseases and the slowing down of aging. However, current explorations of drug targets related to telomere length are usually limited to the context of specific diseases and aging, which limits the discovery of additional drug targets, as researchers prefer to focus on targets that are directly related to specific biological processes.

MR, using genetic variant as an instrumental variable, can reveal the causal effect of exposure on outcome while significantly avoiding confounding factors in observational studies [15]. Proteogenomics focuses on protein expression, regulation, and function at the gene level [16]. The integration of MR with proteogenomics, specifically the use of genetic variants associated with protein expression regulatory loci (pQTL) as exposure variables for MR, has been applied to the identification of potential drug targets for various diseases, such as multiple sclerosis, inflammatory bowel disease, and Parkinson's disease [17–19]. Drug protein targets identified by this integrative research approach can help provide essential clues for precision medicine and drug development. However, as far as we are concerned, investigators have yet to use an approach that integrates large-scale plasma proteomic data and MR to identify plasma drug-protein targets for telomere length.

**Fig. 1:**
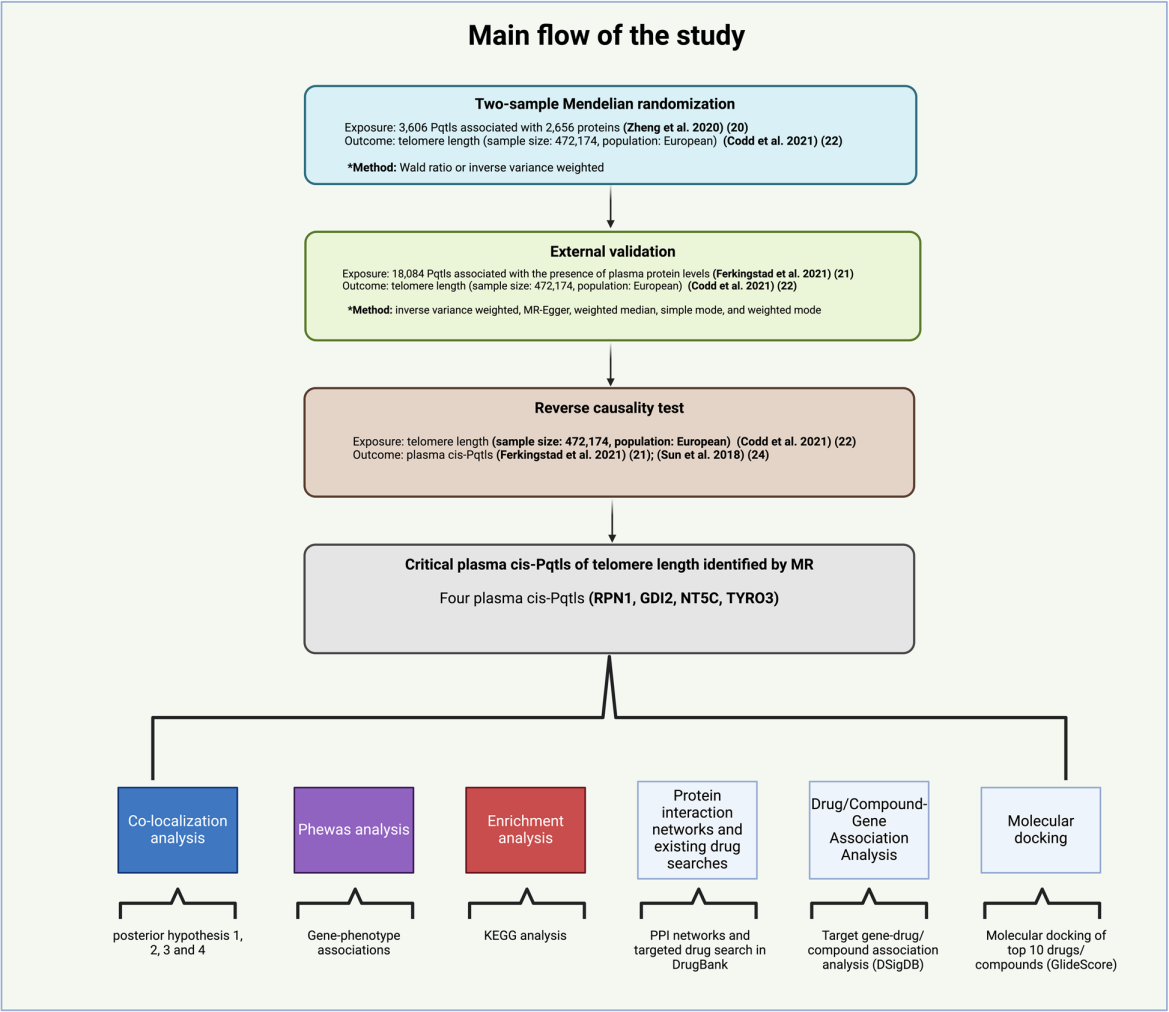
Main flowchart of this MR study (“Created with BioRender.com”)

Therefore, we used two sample-MR, external validation and reverse causality testing to identify key plasma cis-Pqtls for telomere length. To expand our findings, we also performed co-localization analysis, Phenome-wide association studies (PheWAS) analysis, enrichment analysis, protein–protein interaction network construction, existing interventional drug search, drug/compound-gene association prediction, and subsequent molecular docking (Fig. 1).

In summary, this study integrates MR and large-scale pQTL data to identify potential drug targets associated with telomere length regulation at the genetic and gene levels. With this approach, we overcame the limitations of traditional studies in exploring telomere length drug targets, which typically limit the context to different diseases and aging. Also, we opened a systematic pathway for exploring a wide range of applications in telomere biology. This study provides new perspectives and information on telomere length regulation and related drug development. However, there are still significant gaps in translating these findings into practical clinical therapeutic strategies, especially in repetitive validation of drug targets, in-depth exploration of molecular mechanisms, development and testing of drugs, and evaluation of the efficacy and safety of clinical drugs.

## Materials and methods of the study

### Plasma cis-Pqtls data and telomere length dataset

The plasma Pqtls data used for the primary analysis were obtained from an MR study by Zheng et al. that validated 3606 Pqtls associated with 2656 proteins [20]. These data were obtained from five previous GWAS. The plasma Pqtls data used for external validation came from a genome-wide association study (GWAS) performed by Ferkingstad et al., which involved 35,559 Icelanders. Ferkingstad et al. identified 18,084 Pqtls associated with protein levels in plasma [21]. The GWAS dataset for telomere length (sample size: 472,174, population: European) was derived from a GWAS study by Codd et al. [22]. Codd et al. used multiplexed quantitative polymerase chain reaction to determine leukocyte telomere length in a UK biobank of participants between the ages of 45 and 69 years during the period 2006–2010 to assess telomere repeat copy number to single copy gene ratios with stringent quality control and stability assessment [23].

Further, we screened plasma cis-Pqtls. For the extraction of cis-Pqtls for primary analysis, the methodology was consistent with Zheng et al., which involved significant associations (*p* ≤ 5 × 10^–8^), removal of SNPs and proteins within the human Major Histocompatibility Complex (MHC) region, linkage disequilibrium (LD) aggregation (r^2^ < 0.001), pleiotropy, and consistency testing, and cis-pQTL screening within ± 500 kb [20]. Regarding the extraction of cis-Pqtls used for external validation, we adopted the following approach: 1) retain Single nucleotide polymorphisms(SNPs) that were statistically tested to be highly correlated (*p* < 5 × 10^–8^); 2) SNPs with minor allele frequency between 0.01 and 0.99; 3) SNPs adjacent to gene transcription start sites, covering upstream and downstream of the genes 1 MB each; 4) SNPs highly correlated with each other in genetic LD were excluded (r^2^ < 0.001).

### Primary analysis, external validation and reverse causality testing

MR employs genetic variant as an instrumental variable and allows the assessment of the role of protein targets on specific disease or health parameters [15]. In the primary analysis, we used "TwoSampleMR" (https://github.com/MRCIEU/TwoSampleMR) to assess the causal effect of plasma cis-Pqtls on telomere length. If a plasma cis-Pqtls corresponded to a single SNP, the causal effect was assessed using the Wald ratio method; conversely, if plasma cis-Pqtls corresponded to multiple SNPs, the causal effect was assessed using the inverse variance weighted (IVW) MR method. We used the Bonferroni correction to effectively control for the false positive rate due to multiple comparisons (*p* = 0.05/734). For external validation, we extracted SNPs from the deCODE GWAS dataset with significant cis-Pqtls identified by the primary analysis and again validated the association of these protein targets for telomere length, again using Bonferroni correction (*p* = 0.05/11). The increased risk ratio (OR) for telomere length measures the degree of change in risk faced per standard deviation (SD) unit increase in plasma protein levels. In MR, reverse causality testing helps to reduce the effects of confounding and reverse causality, thereby increasing the confidence of causal inferences. Using genetic variation in telomere length as the exposure and genetic variation in plasma cis-Pqtls as the outcome, we performed reverse MR. SNPs for plasma cis-Pqtls were obtained from the GWAS dataset of Sun et al. and the deCODE GWAS dataset [21, 24]. In the reverse MR analysis, we used five methods to assess causal effects: MR-IVW, MR-Egger, weighted median, simple mode, and weighted mode. The primary analysis and external validation dually identified the cis-Pqtls. Identified cis-Pqtls that did not have an inverse causal relationship with the outcome entered the subsequent analysis.

### Co-localization analysis

A Bayesian statistical approach enables the assessment of the joint posterior probability of a specific locus on two phenotypes (e.g., drug target gene and disease). This method provides posterior probabilities about whether two phenotypes share a genetic variable to determine the possibility of co-localization. Bayesian co-localization analyses provide a more comprehensive understanding of the association of genes with multiple phenotypes, thereby strengthening the reliability of causal inferences in MR studies. We performed co-localization analyses of previously identified key plasma cis-Pqtls and telomere length. Genetic variation in plasma cis-Pqtls continues to be derived from the GWAS dataset of Sun et al. and the deCODE GWAS dataset [21, 24]; genetic variant in telomere length is derived from the GWAS dataset of Codd et al. [22]. Using the R package coloc, we calculated posterior hypothesis 4 (PPH4), which is often considered a critical posterior probability in Bayesian co-localization analyses for determining whether two different phenotypes share the same genetic variation. If PPH4 > 0.8, it is considered a potential co-localization, indicating that two different phenotypes are significantly associated with a specific genetic variant [17].

### PheWAS analysis of critical proteins

PheWAS are a broad type of association studys designed to analyze the relationship between one or more gene variants and multiple phenotypes. Using exome sequencing and phenotypic data to generate gene-phenotype association data, the AstraZeneca PheWAS Portal (https://azphewas.com/) provides researchers with an extensive dataset to study potential associations between gene variants and a variety of phenotypes, which can help to identify novel gene-phenotype relationships and to understand gene multifunctionality and multiple effects [25]. The original study assessed associations between rare protein-coding variants and 17,361 binary phenotypes and 1,419 quantitative phenotypes, with phenotypic data derived from detailed phenotypes associated with the medical records of approximately 500,000 participants at UK Biobank [25]. We explored potential horizontal pleiotropy and potential side effects in UK Biobank 470 k (v5) Public by entering crucial plasma cis-Pqtls with a significant threshold set at 2E-9 [26].

### Enrichment analysis

Kyoto Encyclopedia of Genes and Genomes (KEGG) analysis interprets bioinformatics data [27]. It focuses on metabolic pathways, cellular functions, and system-level bioinformation to help researchers understand the functions of genes and their products in specific biological processes [27]. Gene ontology (GO) is a tool used to standardize the description of the function of genes and gene products. It consists of three levels of terminology: molecular function (MF), cellular component (CC), and biological process (BP) [28]. GO enrichment analysis can determine the degree of enrichment of a specific GO term in a defined set of genes, which, in turn, provides information about the function of this set of genes and the biological processes in which they are involved [28]. After inputting key plasma cis-Pqtls, we performed KEGG and GO enrichment analysis using the R package "clusterProfiler."

### Protein–protein interaction network construction and existing drug searches

String is an important database of protein interactions to help understand cellular processes and biological functions (https://cn.string-db.org/). By merging MR-identified drug target proteins with existing identified drug target proteins with telomere lengths and inputting them into String, we mapped the protein–protein interaction (PPI) network (confidence score was set to 0.4, and other parameters were set to default levels) [29–34]. DrugBank is a comprehensive drug database maintained by the University of Alberta that combines detailed drug data with comprehensive drug target information to provide reliable bioinformatics and cheminformatics data (https://go.drugbank.com/). We entered drug target proteins identified by MR and other proteins closely related to those identified by String into DrugBank to explore existing drugs that can modulate these targets.

In addition, we input the drug target proteins identified by MR into GeneMANIA (https://genemania.org/) to generate a protein–protein interaction network map.

### Association analysis of drug target genes with potential drugs and compounds

The Drug Signatures Database (DSigDB) is a database in the Enrichr platform (https://maayanlab.cloud/Enrichr/), mainly used for correlation analysis of drugs and compounds with gene expression [35]. Understanding the effects of drugs on the expression of specific genes is promising for identifying potential therapeutic roles of existing drugs and compounds in new disease areas, thus facilitating new drug applications [35]. We input the critical target genes identified by MR into DSigDB in the Enrichr platform and analyzed their association analysis with different drugs and compounds.

### Molecular docking

For the top 10 drugs/compounds identified by DSigDB's association analysis, we further modeled their interactions with target proteins using molecular docking analysis. For molecular docking experiments, we used Glide (version 92,137), entered the receptor structure and the prepared ligand structure, set the computational parameters (Van der Waals radius scaling factor relative to the standard value of 0.8; charge truncation value of 0.15; torsion control of generality; Glide's Extra Precision mode was enabled; and atom (type assignment using OPLS_2005 force field). In the optimal docking pose, we evaluated the GlideScore, the overall model energy (Emodel), the total energy (E), and the internal energy (internal energy). The GlideScore indicates the mass and strength of interactions in the docking pose, with lower values generally indicating more favorable binding [36–38]. Lower values of Emodel and E indicate that the molecules are more stable and tightly bound, respectively, in the binding state in that pose [38]. Eint is often used to assess a molecule's stability and conformational changes [39].

## Results

### Identification of plasma cis-Pqtls

After qualifying conditions screening, we obtained 734 plasma cis-pQTLs with 738 SNPs for the primary analysis (Additional file 2).

### Four essential drug-target proteins against telomere length

**Fig. 2:**
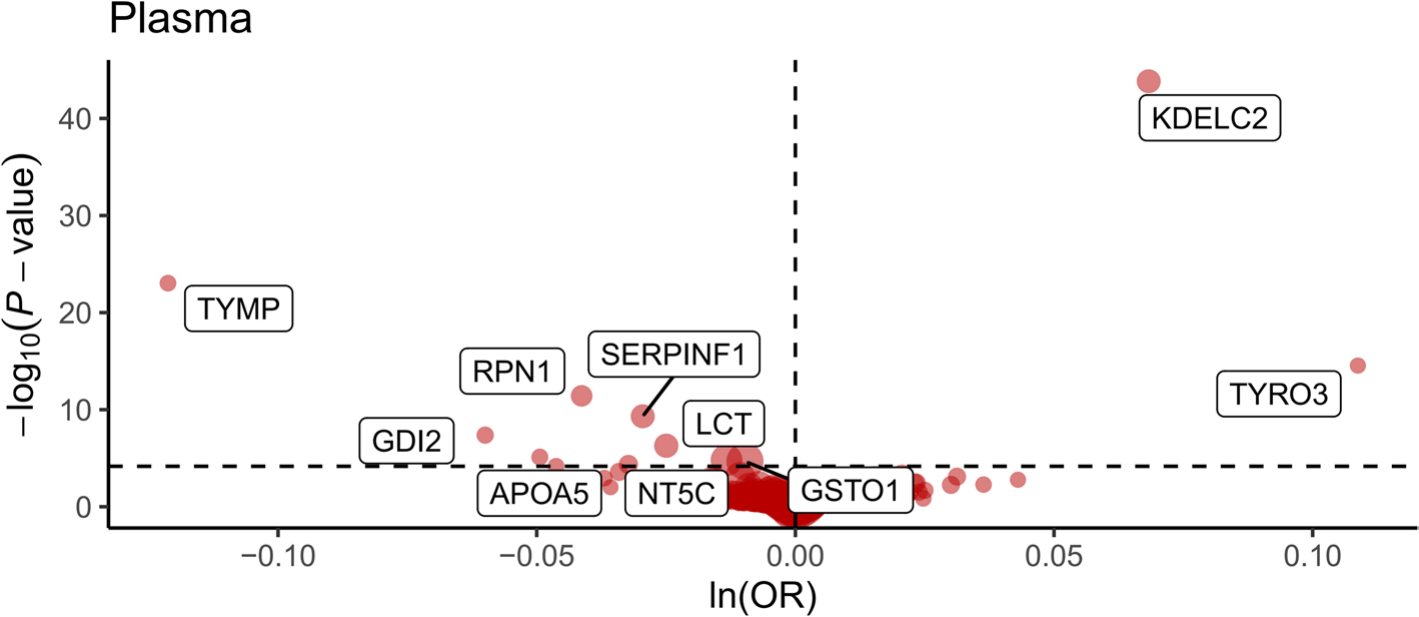
Volcano plots visualizing the main two sample-MR results of the 11 cis-pQTLs and the risk of telomere length. Horizontal black dashed lines across the rows correspond to *P* = 0.05/734. Abbreviations in the graph: ln = natural logarithm

In the primary analysis, we identified 11 drug target proteins with significant causal associations with telomere length (*p* < 0.05/734). According to Wald ratio analysis, nine plasma proteins, APOA5, SERPINF1, RPN1, LCT, TYMP, PSMB1, GDI2, GSTO1, and NT5C, had negative causal associations with telomere length, and two plasma proteins, KDELC2, TYRO3, had positive causal associations with telomere length (Fig. 2) (Table 1). In external validation, five plasma proteins, GDI2, GSTO1, NT5C, RPN1, and TYRO3, remained significantly causally associated with telomere length (*p* < 0.05/11) (Additional file 3). In the reverse causality assay, there was a reverse causal effect of telomere length on GSTO1 (*p* = 0.013) (Additional file 4). As a result of these analyses, we identified GDI2, NT5C, RPN1, and TYRO3 as the four essential drug target proteins for telomere length.

**Table 1:** Causal effects of 11 cis-pQTLs on telomere length

Tissue	Protein	UniProt ID	SNP	Effect allele	OR (95% CI)	*P* value	PVE	F statistics	Author
Plasma	KDELC2	A0A024R3C4;Q7Z4H8	rs74911261	A	1.07 (1.06, 1.08)	1.48E-44	8.61%	310.89	Sun
Plasma	APOA5	A0A0B4RUS7;Q6Q788	rs964184	C	0.95 (0.93, 0.97)	7.66E-06	1.56%	52.29	Sun
Plasma	SERPINF1	A0A140VKF3;P36955	rs62088172	T	0.97 (0.96, 0.98)	4.83E-10	8.69%	314.18	Sun
Plasma	RPN1	P04843	rs2712417	G	0.96 (0.95, 0.97)	3.81E-12	5.57%	194.76	Sun
Plasma	LCT	P09848	rs4988235	A	0.99 (0.98, 0.99)	1.61E-05	23.45%	1011.19	Sun
Plasma	TYMP	P19971	rs131798	G	0.89 (0.87, 0.91)	9.30E-24	1.42%	46.24	Emilsson
Plasma	PSMB1	P20618	rs756519	G	0.98 (0.97, 0.98)	5.19E-07	9.11%	320.88	Emilsson
Plasma	GDI2	P50395	rs2890364	G	0.94 (0.92, 0.96)	4.19E-08	1.75%	57.03	Emilsson
Plasma	GSTO1	P78417;V9HWG9	rs2282326	C	0.99 (0.99, 0.99)	2.12E-05	37.65%	1993.36	Sun
Plasma	TYRO3	Q06418	rs2289743	C	1.11 (1.09, 1.15)	2.88E-15	1.10%	35.56	Emilsson
Plasma	NT5C	Q8TCD5;V9HWF3	rs78625720	A	0.97 (0.95, 0.98)	4.39E-05	3.21%	109.65	Sun

### Results of co-localization analysis

The results of Bayesian co-localization analysis indicated that GDI2 had strong evidence of co-localization with telomere length (coloc.abf-PPH 4 = 9.160649e-01); NT5C, RPN1, and TYRO3 (coloc.abf-PPH 4 < 0.8) did not co-localize with telomere length (Table 2).

**Table 2:** Results of Bayesian co-localization analysis of four essential cis-Pqtls and telomere length

cis-Pqtls	coloc.abf-PPH 0	coloc.abf-PPH 1	coloc.abf-PPH 2	coloc.abf-PPH 3	coloc.abf-PPH 4
TYRO3	8.69E-49	1.32E-19	6.60E-30	1.00E+00	5.96E-08
NT5C	2.17E-19	3.22E-04	6.71E-16	9.96E-01	4.05E-03
RPN1	3.90E-48	1.14E-10	3.41E-38	9.94E-01	5.91E-03
GDI2	4.99E-39	3.29E-14	1.28E-26	8.39E-02	9.16E-01

### PheWAS analysis results

We analyzed the association of GDI2, NT5C, RPN1, and TYRO3 protein expression with numerous binary and continuous phenotypes in the AstraZeneca PheWAS Portal. PheWAS analysis showed that NT5C significantly negatively correlated with the continuous phenotype, cardiometabolicII-associated Q8TCD5 (*p* < 2E -9) (Table 3) (Additional file 5). GDI2, RPN1, and TYRO3 were all not shown to be significantly associated with any of the binary and continuum phenotypes (*p* > 5e-8) (Additional file 1).

**Table 3:** Continuous traits Significantly associated with NT5C using AstraZeneca PheWAS portal

Phenotype	Collapsing model	Phenotypic category	P value	No. samples	Effect size	Effect size standard error	Effect size LCI	Effect size UCI
1072#1906#NT5C#Q8TCD5#OID30294#v1#CardiometabolicII	flexdmg	OLINK Proteomics	2.40E-37	45616	-1.534198395	0.120029789	-1.769458705	-1.298938086
1072#1906#NT5C#Q8TCD5#OID30294#v1#CardiometabolicII	flexnonsynmtr	OLINK Proteomics	2.63E-19	45616	-1.23465192	0.137378543	-1.503916064	-0.965387776
1072#1906#NT5C#Q8TCD5#OID30294#v1#CardiometabolicII	ptv	OLINK Proteomics	1.28E-11	45616	-1.245853126	0.183948058	-1.606394265	-0.885311987
1072#1906#NT5C#Q8TCD5#OID30294#v1#CardiometabolicII	ptv5pcnt	OLINK Proteomics	1.28E-11	45616	-1.245853126	0.183948058	-1.606394265	-0.885311987
1072#1906#NT5C#Q8TCD5#OID30294#v1#CardiometabolicII	ptvraredmg	OLINK Proteomics	2.40E-37	45616	-1.534198395	0.120029789	-1.769458705	-1.298938086
1072#1906#NT5C#Q8TCD5#OID30294#v1#CardiometabolicII	raredmg	OLINK Proteomics	2.38E-27	45616	-1.764394097	0.162755079	-2.083396658	-1.445391536

### Enrichment analysis results

**Fig. 3:**
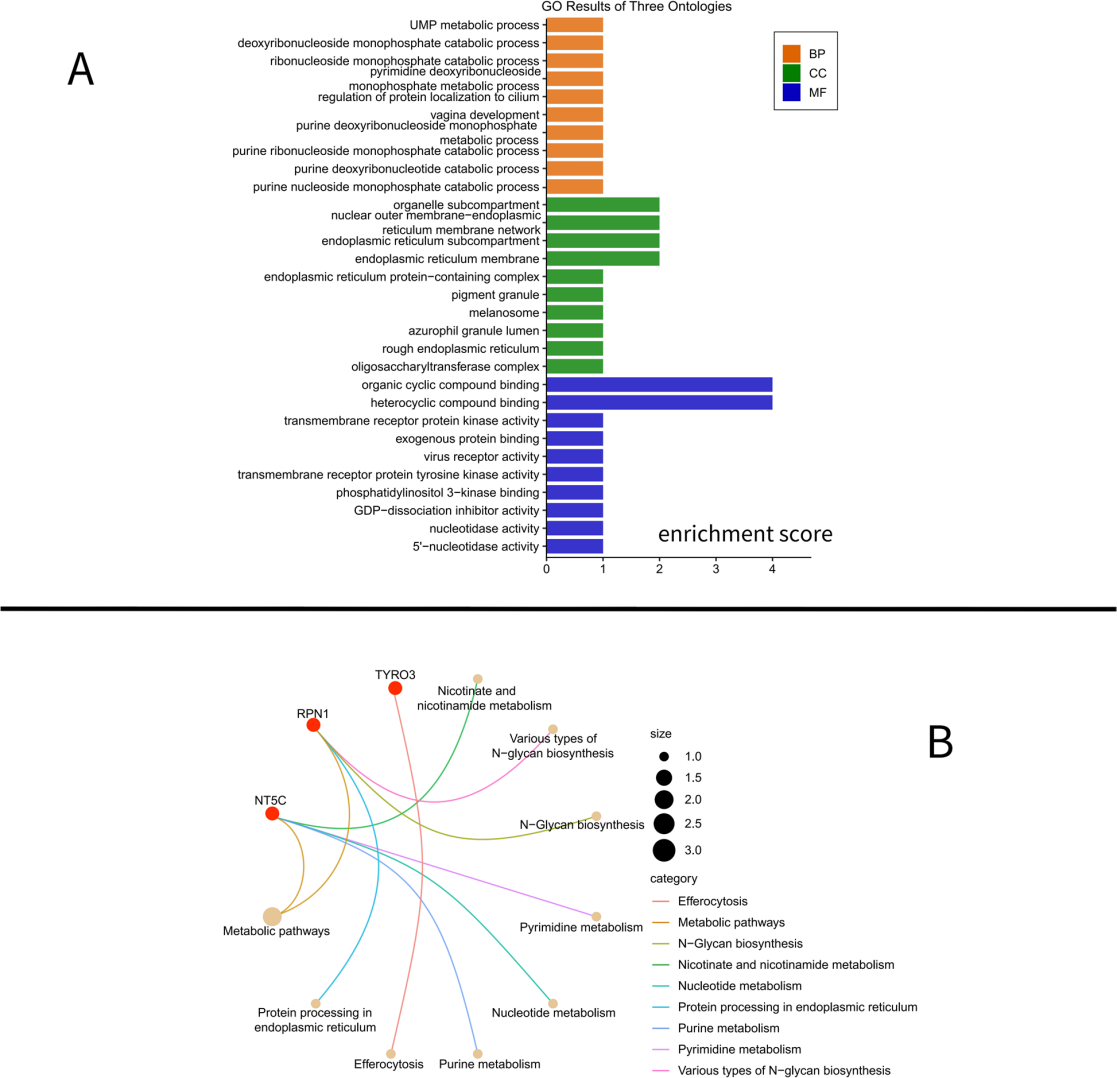
Visualization of enrichment analysis. **A** Visualization of GO enrichment analysis; (**B**) Visualization of KEGG enrichment analysis

In the GO enrichment analysis, concerning the MF category, these target genes were involved in cyclic molecule binding functions, including Heterocyclic Compound Binding and Organic Cyclic Compound Binding. These target genes were involved in endoplasmic reticulum-related cellular components for the CC category. Within the BP category, these drug-targeted genes were closely related to regulating nucleotide metabolism (purines and pyrimidines), UMP metabolism, and protein localization to cilia (Fig. 3A) (Additional file 6).

In KEGG enrichment analysis, three target genes, NT5C, RPN1, and TYRO3, were involved in metabolism-related pathways, such as Nicotinate and Nicotinamide Metabolism, Nucleotide Metabolism, Efferocytosis, and Protein Processing in Endoplasmic Reticulum (Fig. 3B) (Additional file 6).

### Protein interaction network and existing known target drugs

**Fig. 4:**
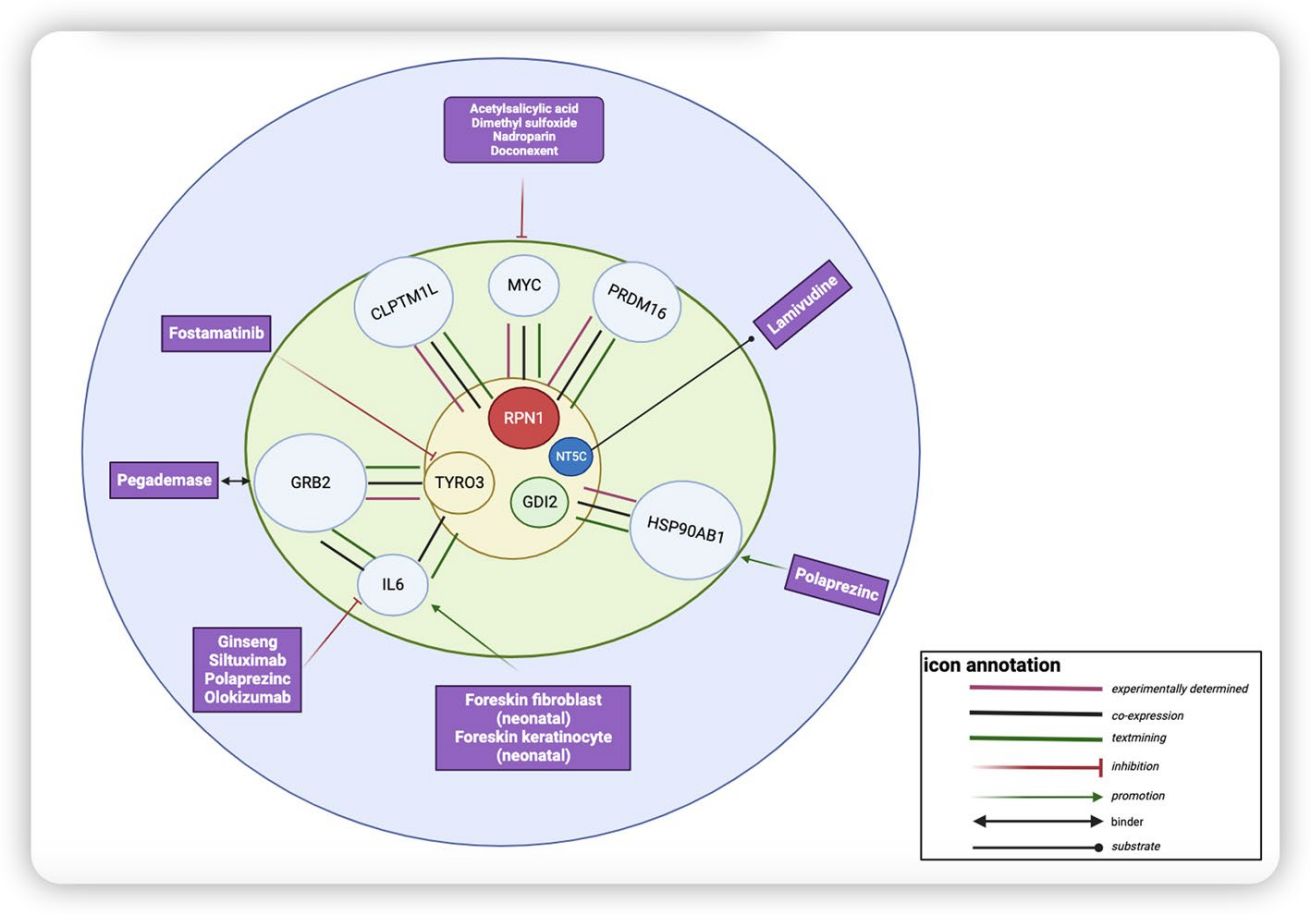
Interaction networks of known target proteins, MR-recognized-critical proteins, and known drugs that can target these targets (“Created with BioRender.com”)

The String-PPI network revealed that three target proteins identified by MR interacted with known drug target proteins for telomere length, of which MYC/CLPTM1L/PRDM16-RPN1, GRB2-IL6-TYRO3, HSP90AB1-GD12 had reliable interactions as having co-expression and experimentally determined (Fig. 4) (Additional file 7). Further, we searched in Drug Bank for known drugs that can target and regulate these proteins. Acetylsalicylic acid, Dimethyl sulfoxide, naproparin, and Doconexent are inhibitors of MYC. Polaprezinc has an HSP90AB1-promoting effect. Ginseng, Siltuximab, Polaprezinc, and Olokizumab were inhibitors of IL6, whereas Foreskin fibroblast and keratinocyte were promoters of IL6. Fostamatinib was an inhibitor of TYRO3 (Fig. 4).

**Fig. 5:**
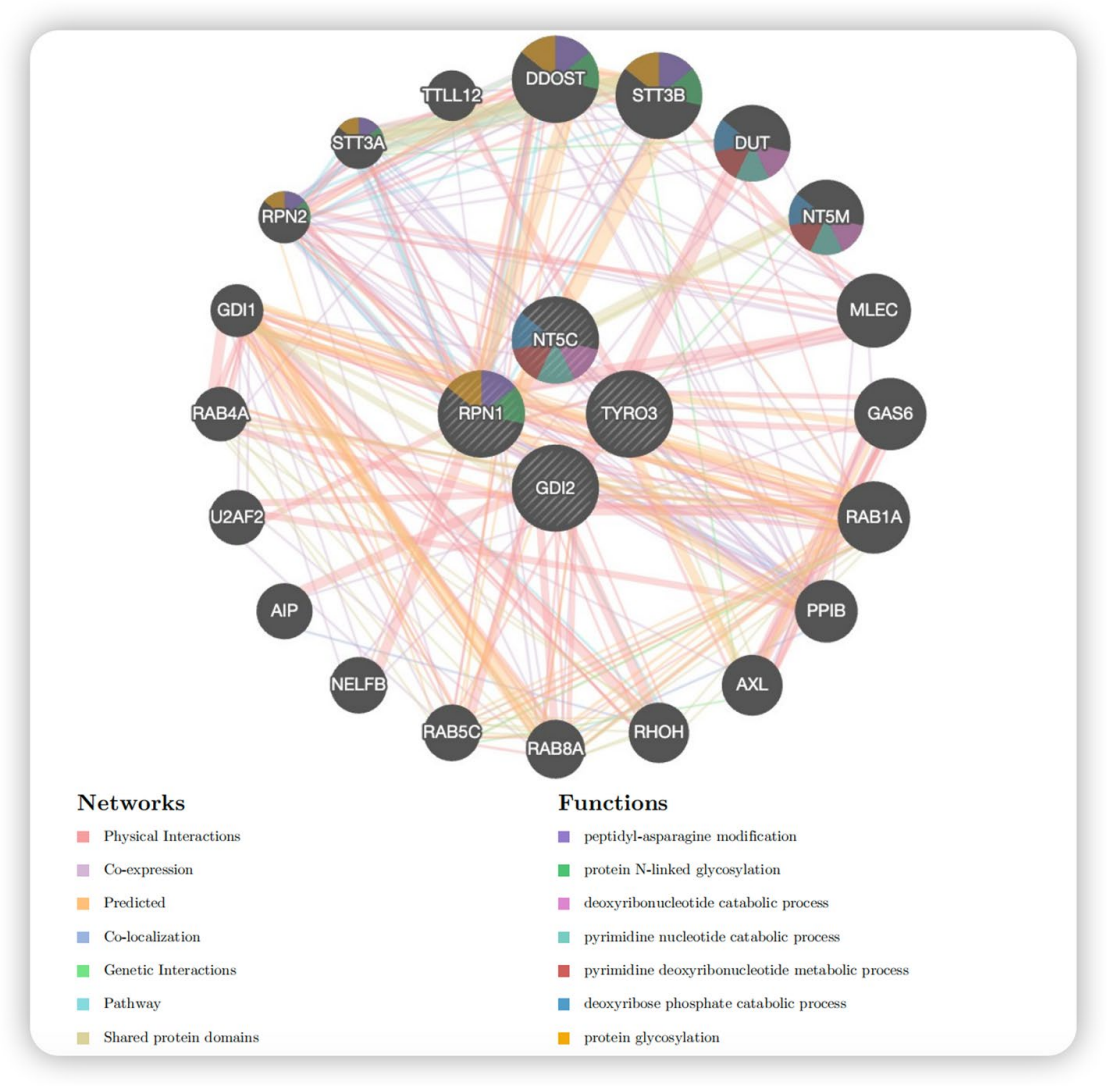
Visualization of the GeneMANIA-PPI network. The colored sectors in each circle indicate the percentage of functional pathways involved in each gene

In the GeneMANIA-PPI network, in addition to the four-drug target proteins recognized by MR, 20 other proteins with interactions with these proteins were included. This PPI network included a total of 311 interacting protein–protein junctions, mainly including physical interaction junctions (77.64%), co-expression junctions (8.01%), prediction junctions (5.37%), and Co-localization junctions (3.63%) (Additional file 8). In addition, the GeneMANIA-PPI network functional analysis results were similar to those of the KEGG analysis, suggesting a high correlation between NT5C, RPN1, and metabolism-related pathways (Fig. 5).

### Drug/compound prediction against MR-identified target genes

Table 4 demonstrates the top ten results of drug/compound-gene association analysis (sorted by Combined Score) using DSigDB on the Enrichr platform (Table 4) (Additional file 9). The association analysis showed that SC-560 and caffeic acid were the drugs/compounds most significantly associated with GDI2; 58–64-0 CTD 00005321 and CI-1033 were the drugs/compounds most significantly associated with TYRO3.

**Table 4:** Top 10 drug/compound candidates predicted using DSigDB

Term	P-value	Adjusted P-value	Old P-value	Old Adjusted P-value	Odds Ratio	Combined Score	Genes
SC-560 MCF7 DOWN	0.00479166	0.071879604	0	0	289.4637681	1545.990778	GDI2
58-64-0 CTD 00005321	0.005588597	0.071879604	0	0	246.5308642	1278.762253	TYRO3
caffeic acid PC3 DOWN	0.005787756	0.071879604	0	0	237.7142857	1224.706508	GDI2
CI-1033 Kinome Scan	0.006185986	0.071879604	0	0	221.8444444	1128.183024	TYRO3
promethazine PC3 DOWN	0.008970245	0.071879604	0	0	151.1515152	712.50441	GDI2
AZD-2171 Kinome Scan	0.009168896	0.071879604	0	0	147.7851852	693.3989792	TYRO3
methylergometrine PC3 DOWN	0.009367518	0.071879604	0	0	144.5652174	675.1928734	GDI2
domperidone MCF7 DOWN	0.009764672	0.071879604	0	0	138.5277778	641.2429069	GDI2
CP-863187 PC3 DOWN	0.009963204	0.071879604	0	0	135.6938776	625.3936139	GDI2
Cabozantinib FDA	0.009963204	0.071879604	0	0	135.6938776	625.3936139	TYRO3

### Results of molecular docking simulations

**Fig. 6:**
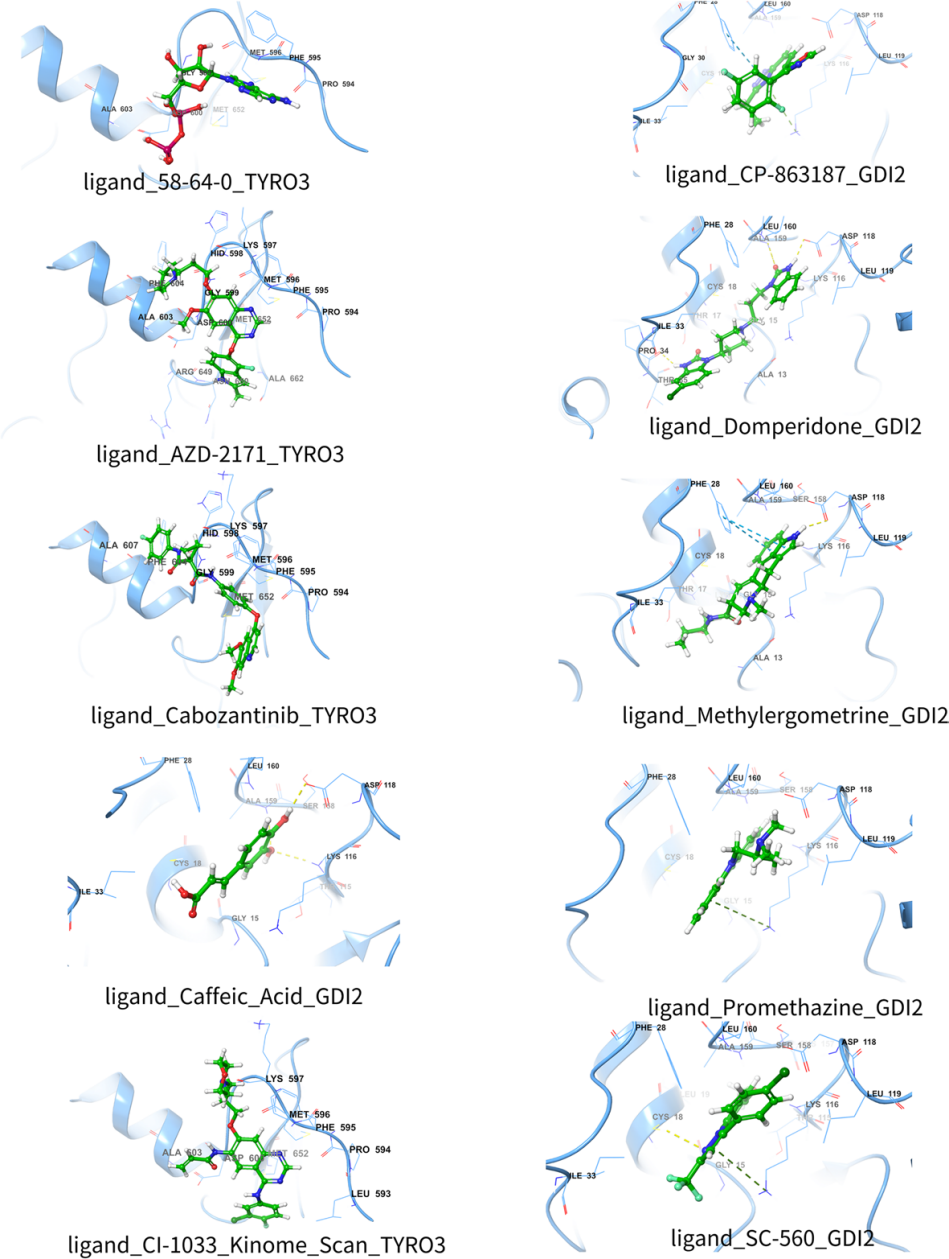
Visualization of molecular docking results of 10 molecules with target proteins

After molecular docking using the Glide molecular docking tool, visualizations of the binding modes of 10 molecules to specific genes or proteins were generated (Fig. 6). 58–64-0 (GlideScore: -7.67658) and Caffeic Acid (GlideScore: -7.25735) were the most tightly bound drugs/compounds, which were docked to TYRO3 and GDI2, respectively. The most tightly bound drugs/compounds, Caffeic Acid and 58–64-0 had low Emodel and E-values, suggesting that they may form a more stable binding site (Supplementary Table)0.58–64-0 had a relatively high Eint (9.99203 kcal/mol), suggesting that 58–64-0 may undergo a significant conformational change upon binding (Additional file 10).

## Discussion

Telomere length is a critical metric associated with multiple health outcomes and aging. Several genes/proteins targeting telomere length play essential roles in the development and progression of certain diseases. Much of the current exploration of telomere-length drug targets has been in the context of specific diseases and aging. However, more information on telomere length-related drug target genes/proteins remains to be explored. To this end, using large-scale plasma cis-Pqtl data and telomere length GWAS datasets, we executed this MR study. Using two-sample MR with external validation and reverse causality testing, we established GDI2, NT5C, RPN1, and TYRO3 as essential proteins for telomere length. Further, we executed Bayesian co-localization analysis, PheWAS analysis, enrichment analysis, protein–protein interaction network construction, known intervening drug search, drug-gene association prediction, and molecular docking. These analyses help to further the biological significance of these drug targets and provide deeper insights for drug development, disease treatment, and individualized medicine.

### Identification of four potential new drug targets for telomere length using MR and large-scale plasma proteomic data

With Pqtls data and disease/health parameter GWAS datasets, MR can be used for drug target identification and validation. The raw Pqtls data for this study's primary analysis contained many pQTLs, covering numerous proteins, allowing for a more comprehensive drug target discovery, i.e., validating known drug targets and providing an opportunity to discover new potential drug targets [20]. In addition, we screened these plasma pQTLs for cis-pQTL, as cis-pQTL are biologically specific by affecting protein levels without affecting mRNA expression or turnover, which helps to identify the effects of protein targets more accurately [40]. In the primary analysis, we identified 11 cis-pQTLs with significant causal associations with telomere length. We performed external validation using pQTL data from deCODE GWAS, excluded six cis-pQTLs and retained the cis-pQTLs that still had significant causal associations with telomere length, GDI2, GSTO1, NT5C, RPN1, and TYRO3. After reversing MR validation, telomere length had a reverse negative causal effect on GSTO1. GSTO1 may regulate telomere length by participating in pathways such as antioxidant and metabolic regulation [41–43]. However, despite the lack of available direct evidence, we hypothesized that telomere length could influence the expression of the GSTO1 gene through telomere position effects [44]. With this in mind, we cautiously excluded GSTO1 as a key drug target for telomere length. To this point, we identified four plasma drug-protein targets, GDI2, NT5C, RPN1, and TYRO3, and implemented subsequent analyses.

### Exploring the genetic link between drug target proteins and telomere length and its biological implications

The results of co-localization analyses showed that GDI2 and telomere length shared the same genetic variants, emphasizing the biological association between GDI2 and telomere length. GDI2 expression is associated with the diagnosis and prognosis of hepatocellular carcinoma, and its signaling is involved in lipid metabolism, extracellular matrix-building pathways, and the tumor microenvironment [45]. Changes in telomere length are involved in cellular senescence, proliferation, and cancer progression [9, 12, 46]. No existing studies demonstrate a direct biological link between GDI2 and telomere length, yet they are both involved in a complex network of tumor development. In the future, further explanation of their common regulatory mechanisms and related molecular pathways in a unified biological context is needed, especially in tumors. NT5C, RPN1, and TYRO3 do not share the same causal genetic variation with telomere length, suggesting that their causal benefits on telomere length do not occur through the same biological pathways. However, the possibility that these target proteins regulate telomere length through different pathways cannot be excluded. The same genetic variants may not necessarily mediate their roles in cell signaling and metabolic pathways. More relevant functional experimental designs and histologic studies, including cytogenetics, proteomics, and metabolomics, should be conducted to reveal the multi-pathway roles of these target genes/proteins in telomere length regulation. PheWAS analysis suggests that NT5C is closely associated with cardiometabolicII-associated Q8TCD5, suggesting that targeting NT5C has a potential role in cardiometabolic pathways. Preclinical and clinical studies need to focus on the potential impact on cardiometabolic health while developing relevant drugs to modulate telomere length. The enrichment analysis results provided information on the MF, CC, BP, and associated pathways of the MR-identified targets, which helped reveal their possible roles in regulating telomere length. Among them, nucleotide metabolism (the metabolic pathway in which MR targets are involved) has been shown to have an essential role in the regulation of telomerase activity and telomere length [47, 48]; the endoplasmic reticulum has also been shown to have an impact on the regulation of telomerase activity, cell proliferation and survival [49, 50].

### Target analysis combining known drug actions with molecular docking potential of new compounds

In existing drug searches against these targets and their associated interacting proteins, the main active ingredient in ginseng, ginsenoside Rg1, has a bi-directional regulatory effect on telomere length [51, 52]. Acetylsalicylic Acid has been associated with telomerase reverse transcriptase, telomerase, and telomere length [53–55]. Acetylsalicylic Acid can inhibit MYC (RPN1 interacting protein) levels in some tumor cells [56]. It has been found that overexpression of the structural region of MYC interacting with TRF1/PIN2 leads to an increase in telomere length [57]. Considering the positive correlation between tumor progression and an increase in telomere length, Acetylsalicylic Acid may be able to inhibit the increase in telomere length by down-regulating the expression of this structural region and thus inhibiting the increase in telomere length. Furthermore, aberrant expression of RPN1, a component of the 26S proteasome motif involved in the cell cycle and protein degradation, may affect tumor proliferation. However, the entire network of effects of acetylsalicylic acid on MYC-RPN1-telomere length is still unknown and needs further investigation, especially in the context of tumors. The IL-6/STAT3 signaling pathway and TYRO3 collectively involve cancer cell survival, proliferation, and resistance to chemotherapeutic agents [58]. The therapeutic role of ginsenoside Rg1 can be explored for inflammatory, immune, and tumor diseases by regulating the IL-6/STAT3-TYRO3-telomere network. Finally, we also used correlation and molecular docking analyses to identify Caffeic Acid and 58–64-0 as two intervening compounds targeting GDI2 and TYRO3, with promise for regulating telomere length. However, the modulatory effect of these two compounds on telomere length by targeting these genes remains to be confirmed by preclinical evidence.

### Biological evidence for the involvement of three protein targets identified by MR in the regulation of telomere length

Although there is a lack of other evidence that these four target proteins are directly involved in regulating telomere length, some indirect evidence can support some of our findings. Dai et al. found that GDI2 is an experimentally confirmed G4-binding protein and that the G4 structure is specialized in DNA molecules closely related to telomere length regulation [59]. This finding indirectly suggests the possibility that GDI2 is involved in regulating telomere structure and influencing telomere function and stability. Further exploration of the mechanisms by which GDI2 affects telomeres and its broader implications for human health is needed. A study performed by Gong et al. used a proteomic approach to identify eight blood circulation proteins, such as GDI2, PSMB4, and PARP1, that had significant causal associations with telomere length (PFDR < 0.05) and good co-localization with telomere length (posterior probability > 0.8) by two-sample MR analysis and co-localization analysis [60]. In addition, they performed mediation analysis and confirmed that some proteins, PARP1, GDI2, and TMEM106A, exerted indirect effects on some diseases, such as prostate cancer, uterine leiomyoma, and idiopathic pulmonary fibrosis through telomere length [60]. Our study used a similar approach and found similar results; for example, we determined that GDI2 has a significant causal association and good co-localization relationship with telomere length. Moreover, we excluded the reverse causal association of telomere length with GDI2 by reverse causality test. An early study performed by Francois et al. confirmed the association of TYRO3 with mammalian telomere dysfunction. TYRO3 is induced to be expressed in response to telomere dysfunction, suggesting they may play a role in telomere-associated cellular processes, such as cell adhesion and growth [61]. This finding affirms the possibility of TYRO3 as a drug target for telomere length, suggesting that TYRO3 expression is a component of the biological pathway for telomere dysfunction. Our findings further suggest that this pathway is associated with changes in telomere length. A previous study identified an SNP in RNP1 (rs60092972) associated with leukocyte telomere length in GWAS of whole genome sequencing data [62]. Our findings are consistent with this study's, revealing that RPN1 (rs2712417) may be modifiable for telomere length. In addition, it is essential to note that RPN1 plays a role in telomere dynamics as part of the proteasome regulatory complex, which mechanistically corroborates the possibility that RPN1 exerts an effect on telomere length [63].

## Limitations

There are also some limitations to this study. Firstly, the GWAS data for leukocyte telomere length was obtained from participants between the ages of 45 and 69 years in the UK Biobank. This GWSA does not limit telomere length measurement to the context of specific diseases and aging, so this could lead to a lack of specificity in the drug targets we screened. Second, in the primary analysis section, all cis-Pqtls were correlated with only 1 SNP, preventing us from performing heterogeneity and pleiotropy analyses of overall causal effects, which may have limited in-depth understanding of multifactorial effects. In the external validation, we only replaced data from plasma cis-Pqtls; we did not replace the outcome GWAS data, as there are no other open source available telomere length GWAS data, which limits the ability of the results to be generalized across different sample sets. Furthermore, although both external validation and primary analysis demonstrated a significant causal effect of TYRO3 on telomere length, the direction of the effect value was missing inconsistently. Therefore, the exact role of TYRO3 in telomere length regulation remains to be further investigated. In the future, MR needs to be utilized to assess the causal effect of more TYRO3-related SNPs on telomere length and for more in-depth experimental validation to identify the regulatory mechanisms. Moreover, although our PheWAS analysis revealed some associations of these MR-identified target proteins with some phenotypes, the specific biological pathways behind these target proteins have not yet been fully elucidated, and it cannot be ruled out that targeting these proteins would adversely affect other protective mechanisms or critical physiological processes in the organism. Therefore, future work needs to deeply explore the biological pathways of these potential drug targets to ensure that drug development does not interfere with the normal function of the organism. Finally, all cis-pQTLs data and telomere length GWAS dataset for the design of this study were derived from European populations. These MR-identified drug targets may not apply to other regional populations and ethnic groups.

## Conclusions

This MR study uniquely identifies critical molecules in telomere length regulation, providing insight into their potential as therapeutic targets. Our work marks a pioneering effort in elucidating the role of four plasma proteins, GDI2, NT5C, RPN1, and TYRO3, in regulating telomere length, providing a new perspective and essential information for the field. These drug targets are promising for addressing cancer and age-related diseases and promoting personalized medicine, as they may modulate telomere length. However, further studies are needed to elucidate the specific mechanisms by which these proteins regulate telomere length. Additionally, we have revealed that 1) GDI2 shares common genetic variation with telomere length and may play an essential role in telomere biology; 2) NT5C may play a role in cardiac diseases; 3) endoplasmic reticulum-associated cellular components and nucleotide metabolism may be involved in the mechanism of telomere length regulation of target genes; and 4) acetylsalicylic acid and ginseng have inhibitory effects on MYC and IL-6 (the interacting proteins of target proteins), respectively; 58–64-0 and caffeic acid have potential as target compounds for telomere length regulation.

In summary, our study provides a foundation for telomere length regulation and targeted drug development. However, more in-depth research is still needed to validate drug targets repeatedly, as well as mechanism exploration and clinical applications.

### Supplementary Information

**Additional file 1: Supplementary Figure 1.** Visualizations of the association of GDI2, RPN1, and TYRO3 with Traits. These visualizations were obtained from the AstraZeneca PheWAS portal.

**Additional file 2: Supplementary Table 1. **Information on all plasma cis-Pqtls included in the primary analysis.

**Additional file 3: Supplementary Table 2. **Main results for the five significant plasma proteins in the external validation.

**Additional file 4: Supplementary Table 3. **Results of reverse causality testing.

**Additional file 5: Supplementary Table 4. **Complete data for four gene-phenotype associations from AstraZeneca PheWAS Portal (https://azphewas.com/).

**Additional file 6: Supplementary Table 5. **Full results of enrichment analysis.

**Additional file 7: Supplementary Table 6. **Data for constructing the String-PPI network.

**Additional file 8: Supplementary Table 7. **Data for the construction of the GeneMANIA-PPI network.

**Additional file 9: Supplementary Table 8. **Full results of drug/compound-gene association analysis, using DSigDB.

**Additional file 10: Supplementary Table 9. **Information on the results of molecule-target proteins generated using the Glide molecular docking tool.

**Additional file 11: Supplementary file. **the STROBE-MR checklist.

## Data Availability

The cis-Pqtls summary data used for the primary analysis are available in the supplementary material of an MR study by Zheng et al. (doi:10.1038/s41588-020-0682-6); the Pqtls data used for the external validation can be obtained from a specific online website request (https://www.decode.com/); and the telomere length GWAS dataset can be obtained from the UK Biobank's open GWAS website (https://gwas.mrcieu.ac.uk/datasets/ieu-b-4879/).
