# Supplementary material for: Insight into telomere regulation: road to discovery and intervention in plasma drug-protein targets
Source: BMC Genomics. 2024 Mar 2;25:231. doi: 10.1186/s12864-024-10116-5 (PMC10909270; doi:10.1186/s12864-024-10116-5)
Supplement: Supplementary file 11 — Additional file 11: Supplementary file. the STROBE-MR checklist. [file 12864_2024_10116_MOESM11_ESM.pdf]

STROBE-MR checklist of recommended items to address in reports of Mendelian randomization studies[1 2]

Item No.	Section	Checklist item	Page No.	Relevant text from manuscript
1	TITLE and ABSTRACT	Indicate Mendelian randomization (MR) as the study's design in the title and/or the abstract if that is a main purpose of the study	1	This study integrated large-scale plasma cis-pQTLs data and telomere length GWAS datasets. We used Mendelian randomization(MR) to identify drug target proteins for telomere length, providing essential clues for future precision therapy and targeted drug development.
	INTRODUCTION			
2	Background	Explain the scientific background and rationale for the reported study. What is the exposure? Is a potential causal relationship between exposure and outcome plausible? Justify why MR is a helpful method to address the study question	2-3	Researchers have recently uncovered the link between specific gene- or protein-related telomere length regulatory effects and certain diseases. A Mendelian randomization(MR) study found that a putative target of simulated metformin, GPD1-induced reduction in HbA 1c, was positively associated with longer leukocyte telomere lengths (11). EGFR enhances telomerase activity (affecting telomere lengths) by potentiating the transcription of TERT, which correlates with the

differentiation grade and prognosis of non-small-cell lung cancer (12). The Shelterin Complex consists of six proteins: TRF1, TRF2, POT1, RAP1, TIN2 and TPP1. Its dysfunction or defects may shorten telomeres and promote aging (13). Upregulation of ETS transcription factors involved in the reactivation of telomerase can diminish the efficacy of BRAF inhibitors in patients with BRAF-mutant pediatric gliomas (14). In the future, an important research direction is to develop specific drug targets to intervene in telomere length, leading to the treatment of diseases and the slowing down of aging. However, current explorations of drug targets related to telomere length are usually limited to the context of specific diseases and aging, which limits the discovery of additional drug targets, as researchers prefer to focus on targets that are directly related to specific biological processes.

The integration of MR with proteogenomics, specifically the use of genetic variants

				associated with protein expression regulatory loci (pQTL) as exposure variables for MR, has been applied to the identification of potential drug targets for various diseases, such as multiple sclerosis, inflammatory bowel disease, and Parkinson's disease (17, 18, 19). Drug protein targets identified by this integrative research approach can help provide essential clues for precision medicine and drug development. However, as far as we are concerned, an investigator has yet to use an approach that integrates large-scale plasma proteomic data and MR to identify plasma drug-protein targets for telomere length.
3	**Objectives**	State specific objectives clearly, including pre-specified causal hypotheses (if any). State that MR is a method that, under specific assumptions, intends to estimate causal effects	3-4	MR, using genetic variation as an instrumental variable, can reveal the causal effect of exposure on outcome while significantly avoiding confounding factors in observational studies (15).<br>The plasma Pqtls data used for the primary analysis were obtained from an MR study by Zheng et al. that validated 3606 Pqtls

			associated with 2656 proteins (20). These data were obtained from five previous GWAS. The plasma Pqtls data used for external validation came from a genome-wide association study (GWAS) performed by Ferkingstad et al. involving 35,559 Icelanders, which identified 18,084 Pqtls that were associated with protein levels in plasma (21). The GWAS dataset for telomere length (sample size: 472,174, population: European) was derived from a GWAS study by Codd et al. (22). Codd et al. used multiplexed quantitative polymerase chain reaction to determine leukocyte telomere length in a UK biobank of participants between the ages of 45 and 69 years during the period 2006–2010 to assess telomere repeat copy number to single copy gene ratios with stringent quality control and stability assessment (23).
	METHODS		
4	**Study design and data sources**	Present key elements of the study design early in the article. Consider including a table listing sources of data for all phases of the study. For each data source contributing to the analysis, describe the following:	

a)	Setting: Describe the study design and the underlying population, if possible. Describe the setting, locations, and relevant dates, including periods of recruitment, exposure, follow-up, and data collection, when available.	4	The plasma Pqtls data used for the primary analysis were obtained from an MR study by Zheng et al. that validated 3606 Pqtls associated with 2656 proteins (20). These data were obtained from five previous GWAS. The plasma Pqtls data used for external validation came from a genome-wide association study (GWAS) performed by Ferkingstad et al. involving 35,559 Icelanders, which identified 18,084 Pqtls that were associated with protein levels in plasma (21). The GWAS dataset for telomere length (sample size: 472,174, population: European) was derived from a GWAS study by Codd et al. (22). Codd et al. used multiplexed quantitative polymerase chain reaction to determine leukocyte telomere length in a UK biobank of participants between the ages of 45 and 69 years during the period 2006–2010 to assess telomere repeat copy number to single copy gene ratios with stringent quality control and stability assessment (23).

b)	Participants: Give the eligibility criteria, and the sources and methods of selection of participants. Report the sample size, and whether any power or sample size calculations were carried out prior to the main analysis	4	The plasma Pqtls data used for the primary analysis were obtained from an MR study by Zheng et al. that validated 3606 Pqtls associated with 2656 proteins (20). These data were obtained from five previous GWAS. The plasma Pqtls data used for external validation came from a genome-wide association study (GWAS) performed by Ferkingstad et al. involving 35,559 Icelanders, which identified 18,084 Pqtls that were associated with protein levels in plasma (21). The GWAS dataset for telomere length (sample size: 472,174, population: European) was derived from a GWAS study by Codd et al. (22). Codd et al. used multiplexed quantitative polymerase chain reaction to determine leukocyte telomere length in a UK biobank of participants between the ages of 45 and 69 years during the period 2006–2010 to assess telomere repeat copy number to single copy gene ratios with stringent quality control and stability assessment (23).

c)	Describe measurement, quality control and selection of genetic variants	4	The plasma Pqtls data used for the primary analysis were obtained from an MR study by Zheng et al. that validated 3606 Pqtls associated with 2656 proteins (20). These data were obtained from five previous GWAS. The plasma Pqtls data used for external validation came from a genome-wide association study (GWAS) performed by Ferkingstad et al. involving 35,559 Icelanders, which identified 18,084 Pqtls that were associated with protein levels in plasma (21). The GWAS dataset for telomere length (sample size: 472,174, population: European) was derived from a GWAS study by Codd et al. (22). Codd et al. used multiplexed quantitative polymerase chain reaction to determine leukocyte telomere length in a UK biobank of participants between the ages of 45 and 69 years during the period 2006–2010 to assess telomere repeat copy number to single copy gene ratios with stringent quality control and stability assessment (23).

d)	For each exposure, outcome, and other relevant variables, describe methods of assessment and diagnostic criteria for diseases	4	The plasma Pqtls data used for the primary analysis were obtained from an MR study by Zheng et al. that validated 3606 Pqtls associated with 2656 proteins (20). These data were obtained from five previous GWAS. The plasma Pqtls data used for external validation came from a genome-wide association study (GWAS) performed by Ferkingstad et al. involving 35,559 Icelanders, which identified 18,084 Pqtls that were associated with protein levels in plasma (21). The GWAS dataset for telomere length (sample size: 472,174, population: European) was derived from a GWAS study by Codd et al. (22). Codd et al. used multiplexed quantitative polymerase chain reaction to determine leukocyte telomere length in a UK biobank of participants between the ages of 45 and 69 years during the period 2006–2010 to assess telomere repeat copy number to single copy gene ratios with stringent quality control and stability assessment (23).

		e)	Provide details of ethics committee approval and participant informed consent, if relevant	NA	NA
5	**Assumptions**		Explicitly state the three core IV assumptions for the main analysis (relevance, independence and exclusion restriction) as well assumptions for any additional or sensitivity analysis	5	MR employs genetic variation as an instrumental variable and allows the assessment of the role of protein targets on specific disease or health parameters (15). In the primary analysis, we used "TwoSampleMR" (https://github.com/MRCIEU/TwoSampleMR) to assess the causal effect of plasma cis-Pqtls on telomere length. If a plasma cis-Pqtls corresponded to a single SNP, the causal effect was assessed using the Wald ratio method; conversely, if plasma cis-Pqtls corresponded to multiple SNPs, the causal effect was assessed using the inverse variance weighted MR method.
6	**Statistical methods: main analysis**		Describe statistical methods and statistics used		
		a)	Describe how quantitative variables were handled in the analyses (i.e., scale, units, model)	5	MR employs genetic variation as an instrumental variable and allows the assessment of the role of protein targets on specific disease or health parameters (15). In the primary analysis, we used "TwoSampleMR" (https://github.com/MRCIEU/TwoSampleMR) to assess the causal effect of plasma cis-Pqtls on telomere length. If a plasma cis-Pqtls corresponded to a single SNP, the causal effect was assessed using the Wald ratio method; conversely, if plasma cis-Pqtls

			corresponded to multiple SNPs, the causal effect was assessed using the inverse variance weighted MR method.
b)	Describe how genetic variants were handled in the analyses and, if applicable, how their weights were selected	4-5	Further, In addition, we screened plasma for cis-Pqtls. For the extraction of cis-Pqtls for primary analysis, the methodology was consistent with Zheng et al., which involved significant associations ($p \leq 5 \times 10^{-8}$), removal of SNPs and proteins within the human Major Histocompatibility Complex (MHC) region, linkage disequilibrium (LD) aggregation ($r^2 < 0.001$), pleiotropy, and consistency testing, and cis-pQTL screening within ±500 kb (20). Regarding the extraction of cis-Pqtls used for external validation, we adopted the following approach: 1) retain Single nucleotide polymorphisms(SNPs) that were statistically tested to be highly correlated ($p < 5 \times 10^{-8}$); 2) SNPs with minor allele frequency between 0.01 and 0.99; 3) SNPs adjacent to gene transcription start sites, covering upstream and downstream of the genes 1MB each; 4) SNPs highly

				correlated with each other in genetic LD were excluded ($r^2 < 0.001$).
		c) Describe the MR estimator (e.g. two-stage least squares, Wald ratio) and related statistics. Detail the included covariates and, in case of two-sample MR, whether the same covariate set was used for adjustment in the two samples	5, 6	MR employs genetic variation as an instrumental variable and allows the assessment of the role of protein targets on specific disease or health parameters (15).<br>In the primary analysis, we used "TwoSampleMR" (https://github.com/MRCIEU/TwoSampleMR) to assess the causal effect of plasma cis-Pqtls on telomere length. If a plasma cis-Pqtls corresponded to a single SNP, the causal effect was assessed using the Wald ratio method; conversely, if plasma cis-Pqtls corresponded to multiple SNPs, the causal effect was assessed using the inverse variance weighted MR method.<br>In the reverse MR analysis, we used five methods to assess causal effects: MR-IVW, MR-Egger, weighted median, simple mode, and weighted mode.
		d) Explain how missing data were addressed	NA	NA
		e) If applicable, indicate how multiple testing was addressed	5	We used the Bonferroni correction to effectively control for the false positive rate due to multiple comparisons (p = 0.05/734).
7	**Assessment of assumptions**	Describe any methods or prior knowledge used to assess the assumptions or justify their validity	6	.We performed co-localization analyses of previously identified key plasma cis-Pqtls

				and telomere length.
8	**Sensitivity analyses and additional analyses**	Describe any sensitivity analyses or additional analyses performed (e.g. comparison of effect estimates from different approaches, independent replication, bias analytic techniques, validation of instruments, simulations)	6	In the reverse MR analysis, we used five methods to assess causal effects: MR-IVW, MR-Egger, weighted median, simple mode, and weighted mode.
9	**Software and pre-registration**			
	a)	Name statistical software and package(s), including version and settings used	5	we used "TwoSampleMR" (https://github.com/MRCIEU/TwoSampleMR) to assess the causal effect of plasma cis-Pqtls on telomere length.
	b)	State whether the study protocol and details were pre-registered (as well as when and where)	NA	NA
	**RESULTS**			
10	**Descriptive data**			
	a)	Report the numbers of individuals at each stage of included studies and reasons for exclusion. Consider use of a flow diagram	25	Figure 1. Main flowchart of this MR study (“Created with BioRender.com”).
	b)	Report summary statistics for phenotypic exposure(s), outcome(s), and other relevant variables (e.g. means, SDs, proportions)	5	The increased risk ratio (OR) for telomere length measures the degree of change in risk faced per standard deviation (SD) unit increase in plasma protein levels.
	c)	If the data sources include meta-analyses of previous studies, provide the assessments of heterogeneity across these studies	5	Further, In addition, we screened plasma for cis-Pqtls. For the extraction of cis-Pqtls for primary analysis, the methodology was consistent with Zheng et al., which involved significant associations ($p \leq 5 \times 10^{-8}$), removal of SNPs and proteins within the human Major Histocompatibility Complex (MHC) region, linkage disequilibrium (LD)

				aggregation ($r^2 < 0.001$), pleiotropy, and consistency testing, and cis-pQTL screening within ±500 kb (20).
		d) For two-sample MR:<br>i. Provide justification of the similarity of the genetic variant-exposure associations between the exposure and outcome samples<br>ii. Provide information on the number of individuals who overlap between the exposure and outcome studies	16	In the primary analysis section, all cis-Pqtls were correlated with only 1 SNP, preventing us from performing heterogeneity and pleiotropy analyses of overall causal effects, which may have limited in-depth understanding of multifactorial effects.
11	**Main results**			
		a) Report the associations between genetic variant and exposure, and between genetic variant and outcome, preferably on an interpretable scale	9	In the primary analysis, we identified 11 drug target proteins with significant causal associations with telomere length ($p < 0.05/734$). According to Wald ratio analysis, nine plasma proteins, APOA5, SERPINF1, RPN1, LCT, TYMP, PSMB1, GDI2, GSTO1, and NT5C, had negative causal associations with telomere length, and two plasma proteins, KDELC2, TYRO3, had positive causal associations with telomere length (**Figure 2) (Table 1)**. In external validation, five plasma proteins, GDI2, GSTO1, NT5C, RPN1, and TYRO3, remained significantly causally associated with telomere length ($p < 0.05/11$) (Supplementary Table 2). In the reverse causality assay, there was a reverse

			causal effect of telomere length on GSTO1 (p = 0.013) (Supplementary Table 3). As a result of these analyses, we identified GDI2, NT5C, RPN1, and TYRO3 as the four essential drug target proteins for telomere length.
b)	Report MR estimates of the relationship between exposure and outcome, and the measures of uncertainty from the MR analysis, on an interpretable scale, such as odds ratio or relative risk per SD difference	5	The increased risk ratio (OR) for telomere length measures the degree of change in risk faced per standard deviation (SD) unit increase in plasma protein levels.
c)	If relevant, consider translating estimates of relative risk into absolute risk for a meaningful time period	5	The increased risk ratio (OR) for telomere length measures the degree of change in risk faced per standard deviation (SD) unit increase in plasma protein levels.
d)	Consider plots to visualize results (e.g. forest plot, scatterplot of associations between genetic variants and outcome versus between genetic variants and exposure)	25-26	**Figure 2**. Volcano plots visualizing the MR results of the main two samples of the 11 cis-pQTLs and the risk of telomere length. Horizontal black dashed lines across the rows correspond to P = 0.05/734. Abbreviations in the graph: ln = natural logarithm; PVE = proportion of variance explained.

12	**Assessment of assumptions**				
		a)	Report the assessment of the validity of the assumptions	16	In the primary analysis section, all cis-Pqtls were correlated with only 1 SNP, preventing us from performing heterogeneity and pleiotropy analyses of overall causal effects, which may have limited in-depth understanding of multifactorial effects.
		b)	Report any additional statistics (e.g., assessments of heterogeneity across genetic variants, such as I^2, Q statistic or E-value)	NA	F statistics
13	**Sensitivity analyses and additional analyses**				
		a)	Report any sensitivity analyses to assess the robustness of the main results to violations of the assumptions	16	In the primary analysis section, all cis-Pqtls were correlated with only 1 SNP, preventing us from performing heterogeneity and pleiotropy analyses of overall causal effects, which may have limited in-depth understanding of multifactorial effects.
		b)	Report results from other sensitivity analyses or additional analyses	NA	NA
		c)	Report any assessment of direction of causal relationship (e.g., bidirectional MR)	9	In the reverse causality assay, there was a reverse causal effect of telomere length on GSTO1 (p = 0.013) (Supplementary Table 3).
		d)	When relevant, report and compare with estimates from non-MR analyses	NA	NA
		e)	Consider additional plots to visualize results (e.g., leave-one-out analyses)	NA	NA
	**DISCUSSION**				
14	**Key results**		Summarize key results with reference to study objectives	12	To this end, using large-scale plasma cis-Pqtl data and telomere length GWAS datasets, we executed this MR study. Using two-sample MR with external validation and reverse causality testing, we established GDI2, NT5C, RPN1, and TYRO3 as essential proteins for telomere length.

15	**Limitations**	Discuss limitations of the study, taking into account the validity of the IV assumptions, other sources of potential bias, and imprecision. Discuss both direction and magnitude of any potential bias and any efforts to address them	16	In the primary analysis section, all cis-Pqtls were correlated with only 1 SNP, preventing us from performing heterogeneity and pleiotropy analyses of overall causal effects, which may have limited in-depth understanding of multifactorial effects.
16	**Interpretation**			
		a) Meaning: Give a cautious overall interpretation of results in the context of their limitations and in comparison with other studies	15	Although there is a lack of other evidence that these four target proteins are directly involved in regulating telomere length, some indirect evidence can support some of our findings. Dai et al. found that GDI2 is an experimentally confirmed G4-binding protein and that the G4 structure is specialized in DNA molecules closely related to telomere length regulation (59). This finding indirectly suggests the possibility that GDI2 is involved in regulating telomere structure and influencing telomere function and stability. Further exploration of the mechanisms by which GDI2 affects telomeres and its broader implications for human health is needed. A study performed by Gong et al. used a proteomic approach to identify eight blood circulation proteins, such as GDI2, PSMB4, and PARP1, that had significant causal associations with telomere length

(PFDR < 0.05) and good co-localization with telomere length (posterior probability > 0.8) by two-sample MR analysis and co-localization analysis (60). In addition, they performed mediation analysis and confirmed that some proteins, PARP1, GDI2, and TMEM106A, exerted indirect effects on some diseases, such as prostate cancer, uterine leiomyoma, and idiopathic pulmonary fibrosis through telomere length (60). Our study used a similar approach and found similar results; for example, we determined that GDI2 has a significant causal association and good co-localization relationship with telomere length. Moreover, we excluded the reverse causal association of telomere length with GDI2 by reverse causality test. An early study performed by Francois et al. confirmed the association of TYRO3 with mammalian telomere dysfunction. TYRO3 is induced to be expressed in response to telomere dysfunction, suggesting they may play a role in telomere-associated cellular processes,

such as cell adhesion and growth (61). This finding affirms the possibility of TYRO3 as a drug target for telomere length, suggesting that TYRO3 expression is a component of the biological pathway for telomere dysfunction. Our findings further suggest that this pathway is associated with changes in telomere length. A previous study identified an SNP in RNP1 (rs60092972) associated with leukocyte telomere length in GWAS of whole genome sequencing data (62). Our findings are consistent with this study's, revealing that RPN1 (rs2712417) may be modifiable for telomere length. In addition, it is essential to note that RPN1 plays a role in telomere dynamics as part of the proteasome regulatory complex, which mechanistically corroborates the possibility that RPN1 exerts an effect on telomere length (63).

	b)	Mechanism: Discuss underlying biological mechanisms that could drive a potential causal relationship between the investigated exposure and the outcome, and whether the gene-environment equivalence assumption is reasonable. Use causal language carefully, clarifying that IV estimates may provide causal effects only under certain assumptions	15	Although there is a lack of other evidence that these four target proteins are directly involved in regulating telomere length, some

indirect evidence can support some of our findings. Dai et al. found that GDI2 is an experimentally confirmed G4-binding protein and that the G4 structure is specialized in DNA molecules closely related to telomere length regulation (59). This finding indirectly suggests the possibility that GDI2 is involved in regulating telomere structure and influencing telomere function and stability. Further exploration of the mechanisms by which GDI2 affects telomeres and its broader implications for human health is needed. A study performed by Gong et al. used a proteomic approach to identify eight blood circulation proteins, such as GDI2, PSMB4, and PARP1, that had significant causal associations with telomere length (PFDR < 0.05) and good co-localization with telomere length (posterior probability > 0.8) by two-sample MR analysis and co-localization analysis (60). In addition, they performed mediation analysis and confirmed that some proteins, PARP1, GDI2, and TMEM106A, exerted indirect effects on

some diseases, such as prostate cancer, uterine leiomyoma, and idiopathic pulmonary fibrosis through telomere length (60). Our study used a similar approach and found similar results; for example, we determined that GDI2 has a significant causal association and good co-localization relationship with telomere length. Moreover, we excluded the reverse causal association of telomere length with GDI2 by reverse causality test. An early study performed by Francois et al. confirmed the association of TYRO3 with mammalian telomere dysfunction. TYRO3 is induced to be expressed in response to telomere dysfunction, suggesting they may play a role in telomere-associated cellular processes, such as cell adhesion and growth (61). This finding affirms the possibility of TYRO3 as a drug target for telomere length, suggesting that TYRO3 expression is a component of the biological pathway for telomere dysfunction. Our findings further suggest that this pathway is associated with changes

				in telomere length. A previous study identified an SNP in RNP1 (rs60092972) associated with leukocyte telomere length in GWAS of whole genome sequencing data (62). Our findings are consistent with this study's, revealing that RPN1 (rs2712417) may be modifiable for telomere length. In addition, it is essential to note that RPN1 plays a role in telomere dynamics as part of the proteasome regulatory complex, which mechanistically corroborates the possibility that RPN1 exerts an effect on telomere length (63).
		c) Clinical relevance: Discuss whether the results have clinical or public policy relevance, and to what extent they inform effect sizes of possible interventions	17	Our work marks a pioneering effort in elucidating the role of four plasma proteins, GDI2, NT5C, RPN1, and TYRO3, in regulating telomere length, providing a new perspective and essential information for the field. These drug targets are promising for addressing cancer and age-related diseases and promoting personalized medicine, as they may modulate telomere length.
17	**Generalizability**	Discuss the generalizability of the study results (a) to other populations, (b) across other exposure periods/timings, and (c) across other levels of exposure	17	Finally, all cis-pQTLs data and telomere length GWAS dataset for the design of this study were derived from European

				populations. These MR-identified drug targets may not apply to other regional populations and ethnic groups.
	OTHER INFORMATION			
18	**Funding**	Describe sources of funding and the role of funders in the present study and, if applicable, sources of funding for the databases and original study or studies on which the present study is based	18	This manuscript received no external funding.
19	**Data and data sharing**	Provide the data used to perform all analyses or report where and how the data can be accessed, and reference these sources in the article. Provide the statistical code needed to reproduce the results in the article, or report whether the code is publicly accessible and if so, where	18	The cis-Pqtls summary data used for the primary analysis are available in the supplementary material of an MR study by Zheng et al. (doi:10.1038/s41588-020-0682-6); the Pqtls data used for the external validation can be obtained from a specific online website request (https://www.decode.com/); and the telomere length GWAS dataset can be obtained from the UK Biobank's open GWAS website (https://gwas.mrcieu.ac.uk/datasets/ieu-b-4879/).
20	**Conflicts of Interest**	All authors should declare all potential conflicts of interest	1	The authors declare no competing interests.

This checklist is copyrighted by the Equator Network under the Creative Commons Attribution 3.0 Unported (CC BY 3.0) license.

1. Skrivankova VW, Richmond RC, Woolf BAR, Yarmolinsky J, Davies NM, Swanson SA, et al. Strengthening the Reporting of Observational Studies in Epidemiology using Mendelian Randomization (STROBE-MR) Statement. JAMA. 2021;under review.

2. Skrivankova VW, Richmond RC, Woolf BAR, Davies NM, Swanson SA, VanderWeele TJ, et al. Strengthening the Reporting of Observational Studies in Epidemiology using Mendelian Randomisation (STROBE-MR): Explanation and Elaboration. BMJ. 2021;375:n2233.
